# Oral microbiome is related to lepidopteran herbivore performance by lignin degradation

**DOI:** 10.1093/ismeco/ycaf229

**Published:** 2025-12-06

**Authors:** Hao-Ran Li, Zhi-Quan Wang, Xiang-Yu Zhang, Yaseen Ullah, Rui Yuan, Jun-Yu Zhao, Xin Xu, Xue Luo, Wei Zhang

**Affiliations:** Jiangsu Key Laboratory for Pathogens and Ecosystems, Jiangsu Engineering and Technology Research Center for Industrialization of Microbial Resources, College of Life Sciences, Nanjing Normal University, Nanjing 210023, China; Jiangsu Key Laboratory for Pathogens and Ecosystems, Jiangsu Engineering and Technology Research Center for Industrialization of Microbial Resources, College of Life Sciences, Nanjing Normal University, Nanjing 210023, China; Jiangsu Key Laboratory for Pathogens and Ecosystems, Jiangsu Engineering and Technology Research Center for Industrialization of Microbial Resources, College of Life Sciences, Nanjing Normal University, Nanjing 210023, China; Department of Animal Ecology, Johann-Friedrich-Blumenbach Institute for Zoology and Anthropology, University of Göttingen, Göttingen 37073, Germany; Centre of Biodiversity and Sustainable Land Use, University of Göttingen, Göttingen 37077, Germany; Jiangsu Key Laboratory for Pathogens and Ecosystems, Jiangsu Engineering and Technology Research Center for Industrialization of Microbial Resources, College of Life Sciences, Nanjing Normal University, Nanjing 210023, China; Jiangsu Key Laboratory for Pathogens and Ecosystems, Jiangsu Engineering and Technology Research Center for Industrialization of Microbial Resources, College of Life Sciences, Nanjing Normal University, Nanjing 210023, China; Jiangsu Key Laboratory for Pathogens and Ecosystems, Jiangsu Engineering and Technology Research Center for Industrialization of Microbial Resources, College of Life Sciences, Nanjing Normal University, Nanjing 210023, China; Jiangsu Key Laboratory for Pathogens and Ecosystems, Jiangsu Engineering and Technology Research Center for Industrialization of Microbial Resources, College of Life Sciences, Nanjing Normal University, Nanjing 210023, China; Nanjing Institute of Environmental Science, Ministry of Ecology and Environment of China, Nanjing 210042, China; Key Laboratory of Pesticide Environmental Assessment and Pollution Control, Ministry of Ecology and Environmental of China, Nanjing 210042, China; Jiangsu Key Laboratory for Pathogens and Ecosystems, Jiangsu Engineering and Technology Research Center for Industrialization of Microbial Resources, College of Life Sciences, Nanjing Normal University, Nanjing 210023, China

**Keywords:** oral microbiota, lepidopteran, lignin degradation

## Abstract

Microorganisms associated with insects play crucial roles in mediating the host plant adaptation of their insect hosts. Although oral microbiota are the primary interface with ingested plant material, we still poorly understand their diversity, their function, and their ecological relationship with insect performance. Here, we investigated the diversity and function of the oral microbiota in two generalist lepidopteran pests (*Spodoptera litura* and *Spodoptera frugiperda*) feeding across three host plants (bok choy, peanut, and maize). Plant species significantly influenced the diversity and composition of oral microbiota in both *S. litura* and *S. frugiperda*. Oral microbial communities from insects feeding on bok choy exhibited significantly higher Sobs richness and Shannon diversity compared to those with peanut or maize plants. Community-level analysis revealed overlapping enriched oral taxa—including *Brevibacterium, Staphylococcus, Microbacterium, Allorhizobium-Neorhizobium-Pararhizobium-Rhizobium, Brachybacterium*, and *Rhodococcus*—that were enriched in both insect species when consuming bok choy. In contrast, they accumulated distinct bacterial taxa emerged when feeding on peanut and maize. Microbial ligninolysis capacity within the oral microbiota showed positive associations with leaf lignin content and herbivore performance. This functional trait primarily associated with *Brevibacterium* and *Rhodococcus* taxa. Accordingly, two isolated strains, *Brevibacterium sedimins* OS20 and *Rhodococcus* sp. OS5 demonstrated effective lignin degradation capacity, achieving 41.01% and 17.62% lignin loss in litter, respectively, after 60 days in microcosm experiments. Overall, host plants shape the diversity and composition of insect oral microbiota. Crucially, microbial ligninolysis capacity and leaf lignin content positively correlated with herbivore performance. This study provides novel insights into the function of oral microbiota in plant–insect interactions, potentially informing the complex multitrophic relationships underlying coevolutionary dynamics.

## Introduction

More than three-quarters of animals are herbivores, which play a pivotal role in regulating both global food webs and the extent of agricultural crop losses [1, 2]. Plants in both natural and agricultural ecosystems utilize various effective defense strategies, including resistance traits, tolerance mechanisms, and escape strategies, to enhance their resilience [3]. In response, herbivores have evolved diverse countermeasures to suppress plant defenses for successful feeding [4]. For instance, herbivorous insects produce effectors within their oral secretions (OS) that are deposited onto plant tissues during feeding to interfere with plant immunity [5–7]. Furthermore, beyond strategies aimed at suppressing plant defenses, herbivorous insects are frequently associated with a range of microbes that facilitate their feeding and nutritional success [8–10].

Herbivorous insects host diverse and abundant bacterial populations within their digestive systems [9, 11]. Certain gut bacteria play a crucial role in adapting these insects to host plants by aiding nutrition, digestion, and detoxification [12]. For example, gut bacteria of the coffee berry borer (*Hypothenemus hampei*) metabolize caffeine, with *Pseudomonas* species identified as key contributors [13]. Similarly, when the specialist silkworm *Bombyx mori* fed on mulberry-derived 1-deoxynojirmycin (DNJ), several bacterial species capable of DNJ degradation, such as *Pseudomonas*, *Staphylococcus*, and *Stenotrophomonas*, were enriched in gut microbiota. Inoculating these enriched microbes into non-specialist insects enhanced host resistance to DNJ and improved their performance [14]. Consequently, the gut bacterial microbiota provides a partial reflection of herbivore performance. However, research on insect-associated bacterial diversity and function predominantly concentrates on the gut community [9, 11], leaving a significant gap in our understanding of the bacterial community within OS. Addressing this gap is particularly critical, as a comprehensive understanding of oral bacterial diversity and function would not only enrich fundamental knowledge of plant–insect interactions, but also unveil novel potential strategies for biocontrol.

Oral microbes inhabit in a dynamic environment, constantly exposed to diverse food particles [15]. Consequently, food sources, particularly host plants, may play a pivotal, albeit unknown, role in directly and indirectly regulating the assembly of oral microbial communities. This influence could occur by modifying the microclimate of the oral cavity or enriching specific bacterial taxa during feeding behavior [5]. Given that oral bacteria are exposed to food earlier than gut bacteria, they may provide earlier indicators of herbivore performance. A detailed understanding of the community-wide impacts of oral bacteria across diverse herbivorous insect-host plant combinations will yield novel insights into the roles of the microbiome in plant–insect interactions.

In this study, to investigate the diversity and function of the oral bacterial community in herbivorous insects feeding on different host plants, two generalist lepidopteran pests, *Spodoptera litura* and *Spodoptera frugiperda*, and three host plants, bok choy, peanut, and maize were included. *Spodoptera litura* and *S. frugiperda* are capable of consuming over 350 species of host plants [16, 17]. The three selected crops, which are globally important, belong to different families and contain distinct leaf metabolites. Furthermore, their production is seriously threatened by lepidopteran pests. Here, we test following hypotheses: (1) host plants exert a significant influence on oral bacterial community composition; (2) oral bacterial community act as indicator of insect performance; and (3) specific taxa within this community play critical ecological roles. We expect that feeding on different host plants will alter the bacterial community structure within herbivore OS, and enrich specific bacterial taxa to improve herbivore performance. Our study deepens our understanding of the role of oral microbe in plant–insect interactions, and facilitates the development of novel strategies for pest control.

## Materials and methods

### Plants and insects

Peanut (Ganhua-5) and maize (Xianyu-335) seeds were obtained from the Ecological Experimental Station of Red Soil, Chinese Academy of Sciences. Bok choy (Shanghai Qing) seeds were purchased from Cangzhou Heshuo Agricultural Technology Co., Ltd. (http://qr.zzxxcx.com/9A8282AFF0815A67).

Eggs of *S. litura* and *S. frugiperda* along with sterile artificial diets were purchased from Henan Jiyuan Baiyun Industry Co., Ltd. (http://www.english.keyunpv.com). The eggs were first surface-sterilized through two consecutive washes with 5 ml of 1% NaClO on sterile filter paper in a petri dish. This involved a brief initial wash followed by a second wash lasting 5 min. Subsequently, the eggs were washed three times with 5 ml of distilled water and dried. The petri dishes were maintained in a climate chamber at 55%–65% relative humidity, 26 ± 2°C under a 16 h photoperiod until the eggs hatching. The newly hatched larvae were reared on the sterile artificial diet in petri dishes until they were used for experiments.

### Greenhouse experiment design

The greenhouse experiment was performed at the Botany Garden of Nanjing Normal University. The soils (0–20 cm layer) were collected from a field in Botany Garden of Nanjing Normal University and homogenized following the removal of visible plant material. A total of 60 pots (17 cm depth and 25 cm diameter) were established. Twenty pots were allocated to each plant species: 10 infected with *S. litura* and 10 infected with *S. frugiperda*. Each pot was planted with either three peanut seedlings, 20 bok choy seedlings, or five maize seedlings. Pots were arranged randomly in a greenhouse maintained at 28 ± 2°C, 55% relative humidity, and a 16 h/8 h (light/dark) photoperoid. Tap water was added as needed. Twenty-one days after planting, bok choy, peanut, and maize plants were infested with *S. litura* and *S. frugiperda* larvae to asses insect performance, as described below.

### Larval performance and OS collection

To assess the growth of *S. litura* and *S. frugiperda* on different host plants, three starved and pre-weighted second-instar larvae were placed on bok choy, peanut, and maize leaves and allowed to feed freely. Infested plants were covered with mesh nets to prevent the larvae from escaping. Larval weights were recorded with a microbalance at 0, 2, 4, 6, and 8 days after the start of the experiment. At the end of the experiment, all insects were used for OS collection.

Larvae of *S. litura* and *S. frugiperda* were first collected from leaves with sterile tweezers. The larvae were kept in sterile plastic petri dishes and transferred to a biological safety cabinet for all subsequent procedures. Next, each larvae was gently immobilized between the thumb and forefinger, followed by the gentle stimulation of the larval mouthpart using a 20 μl pipette tip to induce saliva secretion [18]. The OS from three larvae within each pot were pooled as one individual replicate. The pooled OS were transferred into a sterile 2-ml tube, and centrifuged at 1000 × *g* for 10 min at 4°C to remove leaf debris. The resulting supernatants were collected for microbiome analysis and bacterial isolation.

### DNA extraction, high-throughput sequencing, and bioinformatic analysis

DNA was extracted from OS samples using the FastDNA SPIN Kit (MP Biomedical) following the manufacturer’s instructions. The DNA concentration and integrity were assessed by NanoDrop spectrophotometry (Thermo Fisher Scientific) and electrophoresis, respectively. The amplicon libraries were prepared using universal primers 799F (5′-AACMGGATTAGATACCCK-3′) and 1193R (5′-ACGTCATCCCCACCTTCC-3′). These primers were selected to minimize amplification of chloroplasts and other plant-derived DNA sequences [19]. The reaction system included 1 × Premix Taq DNA polymerase (Takara), 10 ng of DNA templates, and 0.5 μM forward and reverse primers. The amplification was carried out under the following conditions: initial denaturation at 95°C for 3 min, 27 cycles of denaturation at 95°C for 30 s, annealing at 55°C for 30 s, and extension at 72°C for 45 s and a final extension at 72°C for 10 min. Amplicon sequencing libraries were constructed using the MiSeq Reagent Kit v3. Paired-end 300 bp reads were sequenced by Majorbio Bio-Pharm Technology Co., Ltd. on a MiSeq platform (Illumina), following standard protocols.

The raw data were screened and trimmed by the QIIME pipeline, and paired-end sequences were merged using Flash [20, 21]. The clean reads were denoised using DADA2 and viewed as amplicon sequence variants (ASVs) [22]. Taxonomic assignment of ASVs was performed by using the Naive Bayes consensus taxonomy classifier implemented in QIIME2 and the SILVA 16S rRNA database (v138) [23]. Shannon index and Sobs index were used to compare the alpha diversity among treatments. A principal coordinate analysis (PCoA) based on Bray–Curtis dissimilarity was performed to explore patterns of microbial community composition using the Vegan package (version 2.5-6) [24]. Differences in microbial community composition across treatments were determined with permutational multivariate analysis of variance (PERMANOVA, permutation = 999) using the adonis function from the R package (version 3.3.1) [25]. The functional profiles of the gut microbial community were predicted according to the FAPROTAX database (http://www.zoology.ubc.ca/louca/FAPROTAX). Linear discriminant analysis (LDA) of effect size (LEfSe) was applied to the ASV table to identify the differentially abundant taxa [26]. One sample of OS from *S. litura* feeding on maize failed quality control, thus four individual replicates were included in the OS of *S. litura* with maize as host plants. The raw sequencing data were deposited using the SRA service of the GenBank database under the accession number PRJNA1285817.

### Isolation and identification of lignin-degrading oral bacteria

To isolate lignin-degrading bacteria, OS solutions were spread on M9 mineral medium (6.78 g/L Na_2_HPO_4_, 3 g/L KH_2_PO_4_, 0.5 g/L NaCl, 1 g/L NH_4_Cl, 2 mM MgSO_4_, and 0.1 mM CaCl_2_) containing alkalie lignin (0.3%, w/v, Shanghai Yuanye Bio-Technology Co., Ltd.) as sole carbon source after serial 10-fold dilutions. The plates were incubated at 28°C for 10 days in the dark. One representative of each single colony was selected according to the bacterial morphology. A total of 23 bacterial colonies were picked, purified, and stored at −80°C in 20% glycerol until further use.

To determine the lignin degradation capacity of each oral bacterial isolate, the isolates were revived by transferring 100-μl volumes of their freezer stocks into 2 ml of 2 × YT liquid medium (16 g/L tryptone, 10 g/L yeast extract, and 5 g/L NaCl) and cultured overnight at 28°C with shaking (rotary shaker at 180 rpm). Then, 0.5 ml volumes of the revived culture were centrifuged at 5000 × *g* for 8 min. The supernatant was discarded, and the bacterial pellet was resuspended in sterile phosphate buffered saline (PBS) to an OD_600_ of 0.4. A 4 μl aliquot of this suspension was drop-inoculated on the center of an M9 mineral agar plate supplemented with alkalie lignin (0.3%, w/v) and aniline blue (0.1 g/L). After 6 days of incubation at 28°C in the dark, the ratio (D/d) of the decolorization circle diameter (D) to the colony diameter (d) was recorded [27]. Four independent replicates were analyzed.

The identification of bacterial isolates was based on 16S rRNA gene sequence. The bacterial genomic DNA was extracted using a Wizard® Genomic DNA Purification Kit (Promega). The concentration of purified genomic DNA was quantified by a TBS-380 fluorometer (Turner BioSystem Inc.). The 16S rRNA gene was amplified using primer pair 27F (5′-AGAGTTTGATCMTGGCTCAG′-3′) and 1492R (5′-TACGGYTACCTTGTTACGACTT-3′). The PCR reactions (50 μl) contained 2 μl of bacterial DNA, 25 μl of master mix, 1 μl of each of the forward and reverse primers, and 21 μl of deionized water. The PCR was run as follows: initial denaturation at 94°C for 5 min, 30 cycles of denaturation at 94°C for 30 s, annealing at 55°C for 30 s, extension at 72°C for 1 min, and a final extension at 72°C for 10 min. The PCR products were sequenced by Sanger sequencing (Tsingke). The obtained 16S rRNA sequences were blasted against the National Center for Biotechnology Information (NCBI) database to identify homologous sequences, and the closest match was identified. The 16S rRNA sequences have been deposited in the GenBank database under accession numbers PV991484–PV991506. Phylogenetic trees were constructed using maximum likelihood with the obtained sequences.

To determine the exact lignin degradation efficiency of OS bacteria, we used the Prussian blue assay to detect the lignin content in bacterial cultures. To this end, OS bacteria were incubated overnight at 28°C using 2 × YT liquid medium, followed by centrifugation at 5000 × *g* for 8 min. After centrifugation, the bacterial suspensions were adjusted to an OD_600_ of 0.4 with sterile PBS, and then 100 μl of suspension was added to 5 ml of M9 mineral medium supplemented with alkalie lignin (0.3%, w/v). After 10 days of cultivation at 28°C with shaking (rotary shaker at 180 rpm), the bacterial pellet and cultural medium were separated and collected for bacterial colony-forming units (CFUs) and lignin content determination, respectively.

### Determination of bacterial CFUs and lignin content in culture medium

To determine the bacterial CFUs, the bacterial pellet was resuspended with sterile PBS and spread on 2 × YT agar plates after serial 10-fold dilutions. The plates were maintained at 28°C for 3 days in the dark, and numbers of colonies were counted. Results were expressed as CFU per ml of medium culture. Three independent replicates were analyzed.

The residual lignin in the culture medium was completely dissolved by adjusting the pH to 12.5 with 2 M NaOH. A total of 1.5 ml of the dissolved lignin solution was mixed with 100 μl of 8 mM K_3_Fe(CN)_6_ and 100 μl of 0.1 M FeCl_3_, incubated for 5 min at room temperature, and monitored by absorbance at 700 nm to determine the concentration of lignin [28]. Three independent replicates were analyzed.

### Leaf chemistry analyses

Leaf samples of bok choy, peanut, and maize were harvested 21 days after planting to determine soluble sugar, protein, cellulose, and lignin content. The leaves were divided into two portions: one fresh sample was used for soluble sugars and protein analysis, while the rest was used for the lignin and cellulose determination.

Soluble sugars and protein analysis: Fresh leaf tissue was extracted with 80% ethanol. Following incubation at 80°C for 20 min, samples were centrifuged at 12 000 × *g* for 20 min, and the supernatants were collected. The pellets were re-extracted twice using the same procedures. All supernatants were pooled for subsequent sucrose, glucose, and fructose quantification. Soluble sugar and protein concentrations were detected with assay kits (Nanjing Jiancheng Bioengineering Institute) [29]. Protein content was quantified by the Bradford method [30]. Five independent replicates were analyzed.

Lignin and cellulose analysis: Leaf samples were dried at 80°C to constant weight, pulverized, and passed through a 40-mesh sieve. The cellulose and lignin contents were determined using the corresponding assay kits according to the manufacturer’s instructions (Nanjing Tibco Testing Technology Co., Ltd.). Five independent replicate samples were similarly analyzed.

### Microcosm experiment design

Bok choy leaves were collected and dried to a constant weight at 80°C. The resulting litter fragments were filtered through 2-mm sieves. The decomposition experiment followed a modified protocol [31]. Briefly, 1 g of dried litter was evenly distributed onto a cellophane sheet placed over 10 ml of 1.5% agarose gel in a 90-mm petri dish. The cellophane facilitated the subsequent litter retrieval. Two milliliters of either microbial inoculum or sterile PBS (control) were evenly sprayed onto the litter in each plate. To prevent contamination, petri dishes were placed inside sterile 1-L plastic container, with two dishes per container. Container were randomly arranged and incubated in the dark at 25°C. The microorganisms on the litter fragments were observed by scanning electron microscopy (SEM, JEOL) according to a previous study [32]. Litter samples were collected after 30 and 60 days of incubation. Samples were then dried to constant weight at 80°C and weighted. Lignin content was determined as described above (See: Leaf chemistry analyses). The experiment was performed in five independent replicates. Litter lignin loss (%) was calculated using the following formula:


$$ \mathrm{Litter}\kern0.17em \mathrm{lignin}\kern0.17em \mathrm{loss}\;\left(\%\right)=\left(\left[{\mathrm{M}}_{\mathrm{i}}\times{\mathrm{L}}_{\mathrm{i}}\right]\hbox{--} \left[{\mathrm{M}}_{\mathrm{p}}\times{\mathrm{L}}_{\mathrm{p}}\right]\right)/\left({\mathrm{M}}_{\mathrm{i}}\times{\mathrm{L}}_{\mathrm{i}}\right)\times 100, $$

where M_i_ and M_p_ are the initial and post-incubation litter masses, respectively, and L_i_ and L_p_ are initial and post-incubation lignin concentrations, respectively.

### Whole-genome sequencing of *B. sedimins* OS20 and *Rhodococcus* sp. OS5

Genomic DNA from *B. sedimins* OS20 and *Rhodococcus* sp. OS5 was extracted using a Wizard® Genomic DNA Purification Kit (Promega). The concentration of the purified genomic DNA was quantified with a TBS-380 fluorometer (Turner BioSystem Inc.). Genome sequencing was performed on the Illumina Hiseq and PacBio platforms, with a shotgun library of 400 bp insertion size. Quality control of raw data was performed in Fastp v0.20.0. Genomes were assembled using Unicycler [33]. Gene annotation was carried out with Prodigal v2.6.3. Databases, including NR, Swiss-Prot, Pfam, EggNOG, GO, and KEGG, were used for functional annotation of the predicted coding genes. The rRNA gene sequences were identified using Barrnap v0.9.

### RNA extraction and qRT-PCR analysis

*Brevibacterium sedimins* OS20 and *Rhodococcus* sp. OS5 were incubated overnight at 28°C using 2 × YT liquid medium, followed by centrifugation at 5000 × *g* for 8 min. After centrifugation, the bacterial suspensions were adjusted to an OD_600_ of 0.4 with sterile PBS, and then 100 μl of suspension was added to 5 ml of M9 mineral medium supplemented with glucose (2%, w/v) or alkalie lignin (0.3%, w/v). After 10 days of cultivation at 28°C with shaking (rotary shaker at 180 rpm), the bacterial pellet was separated and collected for RNA extraction. The 16S rRNA gene was used as reference gene to normalize the expression of selected gene using the 2^−ΔΔCt^ method. The experiment was performed with four individual replicates and three technological replicates. The selected genes and primers are listed in Table S1.

### Statistical analysis

Statistical analyses were performed using SPSS (version 22) and R (version 3.3.1). Two-sided, unpaired Student’s *t*-test was used to determine variation in lignin degradation (%) and bacterial CFUs. Two-sided, paired Student’s *t*-test was used to determine variation in microbial α-diversity (Sobs index and Shannon index) between insect species. ANOVA with Tukey’s HSD test was used to determine the significant differences in larval weight, microbial α-diversity, relative abundance of ASV61 and ASV123, bacterial lignin degradation capacity (D/d), and lignin loss in litter (%) among treatments. PERMANOVA based on Bray–Curtis distance with 999 permutations was used to determine microbial β-diversity. Kruskal–Wallis (KW) sum-rank test and the absolute LDA score (>3.0) were used to identify key biomarker species across different treatments. Linear regression was used to assess the linear relationship between leaf chemistry properties with ligninolysis abundance and larval weight. Redundancy analysis (RDA) was used to clarify the relationship between bacterial genera and leaf chemistry properties. RDA was performed using the R package “vegan.”

## Results

### Performance of lepidopteran herbivore on different host plants

**Figure 1:**
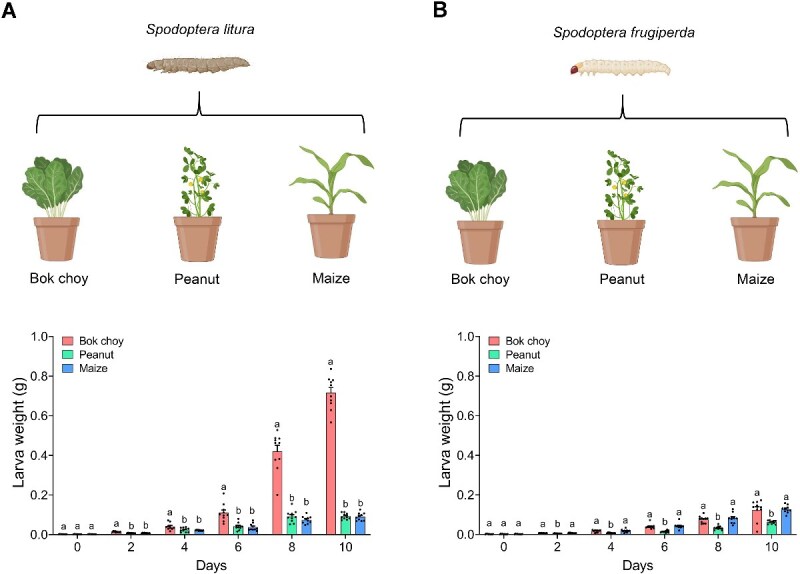
Larval performance of *S. litura* and *S. frugiperda* on different host plants. (A) Larval weight of *S. litura* when feeding on bok choy, peanut, and maize plants. (B) Larval weight of *S. frugiperda* when feeding on bok choy, peanut, and maize plants. Data are presented as means ± SEM (*n* = 10 biological replicates). Different letters denote significant differences (*P* < .05, ANOVA followed by Tukey’s HSD test). Cartoons were created with BioRender (https://BioRender.com).

We conducted pot experiments with six lepidopteran insect-plant combinations (two insects: *S. litura* and *S. frugiperda*; three plant species: bok choy, peanut, and maize) to investigate the insect performance across host plants. Although both *S. litura* and *S. frugiperda* were generalist herbivores, they exhibited distinct host plant preferences. *Spodoptera litura* larvae reared on bok choy achieved significantly higher biomass than those feeding on peanut or maize, accumulating 7.70- and 7.85-fold more biomass, respectively, at 10 days (ANOVA, *F* = 517.8, *P* < .05; Fig. 1A). *Spodoptera litura* larval biomass did not differ between peanut and maize feeding groups (ANOVA, *P* > .05). In contrast, *S. frugiperda* larvae accumulated similar biomass on bok choy and maize (ANOVA, *P* > .05), both of which were significantly higher than biomass attained on peanut seedlings (ANOVA, *F* = 17.23, *P* < .05; Fig. 1B).

### Plants species affect the oral bacterial community of lepidopteran herbivores

**Figure 2:**
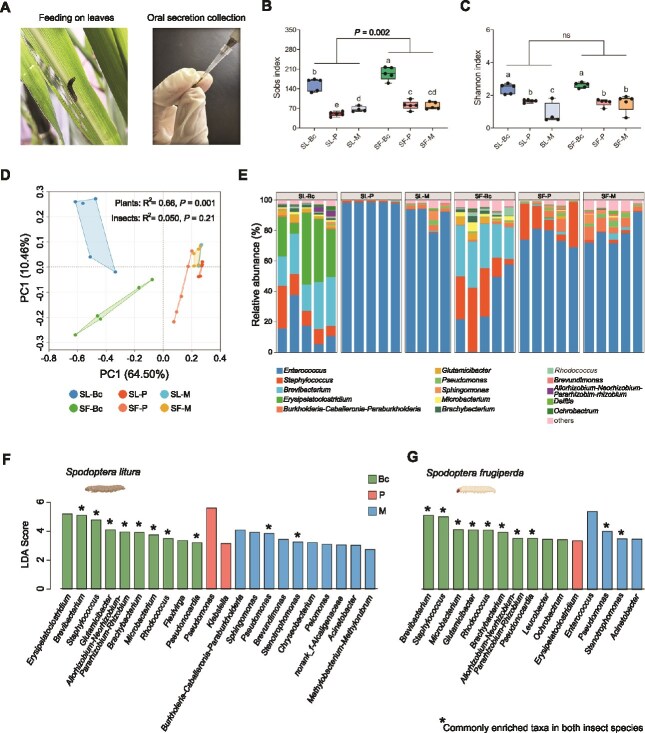
Plants species affect the oral bacterial communities of *S. litura* and *S. frugiperda*. (A) Images depicting larval feeding on leaves and OS collection. Sobs (B) and Shannon (C) index of the bacterial community in OS samples from SL-Bc, SL-P, SL-M, SF-Bc, SF-P, and SF-M treatments. Data are presented as means ± SEM (*n* = 4–5 biological replicates). Different letters denote significant differences (*P* < .05, ANOVA followed by Tukey’s HSD test). Asterisks denote statistically significant differences via a two-sided, paired Student’s *t*-test (^**^P < .01). ns indicates not significant via a two-sided, paired Student’s *t*-test (*P* > .05). (D) PCoA based on Bray–Curtis distance calculated from the relative abundance of bacterial ASVs in OS samples from SL-Bc, SL-P, SL-M, SF-Bc, SF-P, and SF-M treatments. Statistical significance was assessed by permutational multivariate analysis of variance (PERMANOVA). (E) Relative abundance of bacterial genera in OS samples from SL-Bc, SL-P, SL-M, SF-Bc, SF-P, and SF-M treatments. LDA of effect size (LEfSe) identifying bacterial genera enriched in OS samples of *S. litura* (F) and *S. frugiperda* (G) feeding on bok choy, peanut, and maize plants. Asterisks denote the commonly enriched bacterial genera in both insect species when feeding on the same plant species. SL-Bc: *S. litura* feeding on bok choy, SL-P: *S. litura* feeding on peanut, SL-M: *S. litura* feeding on maize, SF-Bc: *S. frugiperda* feeding on bok choy, SF-P: *S. frugiperda* feeding on peanut, and SF-M: *S. frugiperda* feeding on maize.

OS samples were collected from *S. litura* and *S. frugiperda* larvae during feeding on bok choy, peanut, or maize plants for microbiome analysis (Fig. 2A). The oral bacterial communities of both *S. litura* and *S. frugiperda* exhibited higher Sobs and Shannon index when they feeding on bok choy, compared to peanut and maize (ANOVA, Sobs: *F* = 57.33, *P* < .05; Shannon: *F* = 14.28, *P* < .05; Fig. 2B and C). Insect species significantly affected the Sobs index of oral bacterial communities (Paired Student’s *t*-test, *P* = .002), but not the Shannon index (Paired Student’s *t*-test, *P* = .14). PCoA revealed that host plant species have a significant impact on OS bacterial community composition (PERMANOVA, R^2^ = 0.66, *P* = .001) than insect species (PERMANOVA, R^2^ = 0.050, *P* = .21; Fig. 2D). Communities from larvae feeding on bok choy were significantly separated from those feeding on peanut and maize. When comparing the OS microbial communities of *S. litura* and *S. frugiperda* fed on same host plants, we found that two species exhibited distinct clustering patterns in OS microbial community when they fed on bok choy and peanut, but not on maize (Fig. S1). At the community-level, *Enterococcus*, *Staphylococcus*, *Brevibacterium*, *Erysipelatoclostridium*, and *Burkholderia*-*Caballerinia*-*Parabukholderia* were the top five most abundant genera (Fig. 2E). LefSE analysis identified *Brevibacterium*, *Staphylococcus*, *Microbacterium, Allorhizobium-Neorhizobium-Pararhizobium-Rhizobium*, *Brachybacterium*, and *Rhodococcus* as enriched taxa in both *S. litura* and *S. frugiperda* associated with bok choy feeding. In contrast, *S. litura* and *S. frugiperda* harbored distinct enriched oral bacterial taxa when feeding on peanut and maize (Fig. 2F and G).

### Oral microbial Ligninolysis function is positively associated with lepidopteran herbivore performance

**Figure 3:**
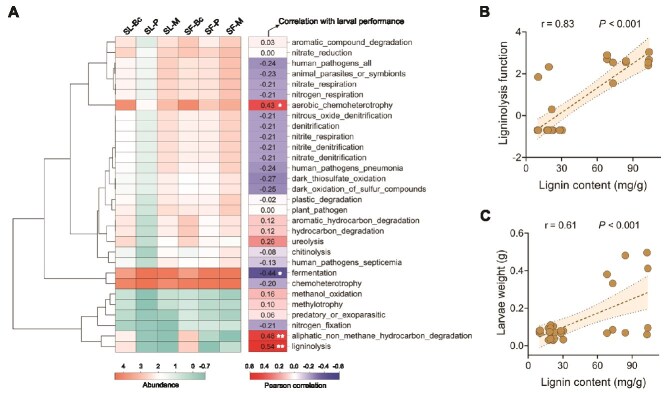
Ligninolysis function within the oral bacterial community is positively associated with leaf lignin content and herbivore performance. (A) Microbial functional abundance in relation to larval weight. Asterisks indicate statistically significant differences (^*^P < .05, ^**^P < .01). (B) Leaf lignin in relation to microbial ligninolysis function abundance. (C) Leaf lignin in relation to larval weight.

We predicted functional profiles of the oral microbiota using the FAPROTAX database and correlated these functional traits with larval performance. The functional traits, aerobic_chemohetero-trophy, aiphatic_non_methane_hydrocarbon_degradation, and ligninolysis were significantly positively correlated with larval biomass, with ligninolysis exhibited a stronger correlation (*r* = 0.54, *P* = .0027; Fig. 3A). The ligninolysis function was enriched in larvae when feeding on bok choy compared to those feeding on peanut and maize. Consistently, bok choy leaves contained a higher lignin concentrations than peanut and maize leaves (ANOVA, *F* = 63.34, *P* < .05; Fig. S2). Leaf lignin content was also significantly positively correlated with both microbial ligninolysis capacity (*r* = 0.83, *P* < .001, Fig. 3B) and larval biomass (*r* = 0.61, *P* < .001; Fig. 3C). In contrast, leaf soluble sugars and protein content showed no significant relationship with larval biomass (Fig. S3). Together, these results indicate that leaf lignin content and oral microbial ligninolysis function are associated with enhanced herbivore performance.

### *Brevibacterium* and *Rhodococcus* taxa contribute to ligninolysis

**Figure 4:**
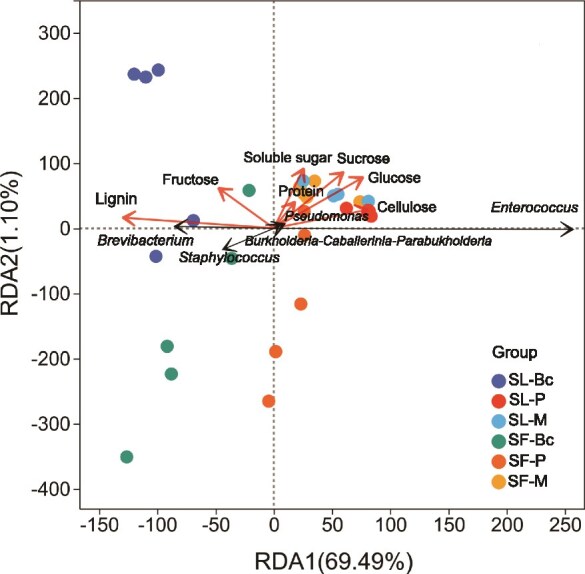
RDA displaying the relationships between OS bacterial community composition and leaf chemistry properties. Black arrows represent the top five most abundant bacterial genera; red arrows indicate leaf chemistry properties.

Consistent to the results of LefSE analysis, the genera *Brevibacterium* and *Staphylococcus* were associated with the oral bacterial communities of insects fed on bok choy. Leaf lignin content showed a positive correlation with the relative abundance of *Brevibacterium* but a negative correlation with that of *Enterococcus*. In contrast, the relative abundance of *Enterococcus* was positively associated with the leaf cellulose content (Fig. 4). Although *Staphylococcus* taxa were enriched in the OS of both *S. litura* and *S. frugiperda* fed on bok choy, while it was also enriched in OS of *S. frugiperda* fed on peanut (Fig. 2E). Crucially, the microbial ligninolysis function in the OS of *S. frugiperda* fed on peanut was much lower than in the OS of *S. litura* and *S. frugiperda* fed on bok choy (Fig. 3A). This discrepancy suggests that *Staphylococcus* enrichment is not directly linked to ligninolysis.

**Figure 5:**
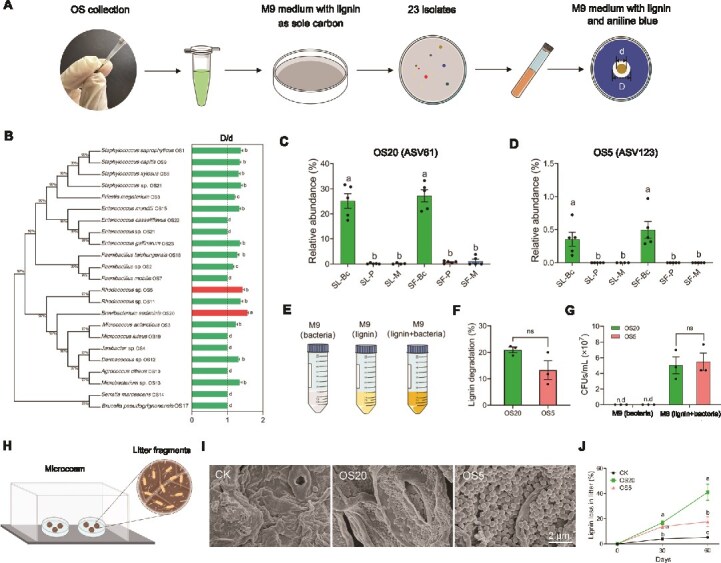
*Brevibacterium sedimins* OS20 and *Rhodococcus* sp. OS5 degrade lignin. (A) Isolation of OS bacterial strains with lignin-degrade capacity. A total of 23 strains is obtained. (B) Capacity of 23 strains to degrade lignin on M9 plates. Phylogenetic trees were constructed using maximum likelihood with 16S rDNA sequences. Data are presented as means ± SEM (*n* = 3 biological replicates). Different letters denote significant differences (*P* < .05, ANOVA followed by Tukey’s HSD test). (C and D) The relative abundance of ASV61 and ASV123 in OS samples. Data are presented as means ± SEM (*n* = 4–5 biological replicates). Different letters denote significant differences (*P* < .05, ANOVA followed by Tukey’s HSD test). (E) *Brevibacterium sedimins* OS20 and *Rhodococcus* sp. OS5 were cultured in M9 liquid medium with or without alkalie lignin addition. (F) Lignin degradation of *B. sedimins* OS20 and *Rhodococcus* sp. OS5. Data are presented as means ± SEM (*n* = 3 biological replicates). ns indicates not significant via a two-sided, unpaired Student’s *t*-test (*P* > .05). (G) Bacterial CFUs of *B. sedimins* OS20 and *Rhodococcus* sp. OS5. Data are presented as means ± SEM (*n* = 3 biological replicates). ns indicates not significant via a two-sided, unpaired Student’s *t*-test (*P* > .05). n.d indicates not detected. (H) Microcosm experimental design. Two petri dishes were placed inside sterile 1-L plastic container. (I) Representative SEM images showing dense bacterial aggregation on the litter fragments. (J) Lignin loss in litter after *B. sedimins* OS20 and *Rhodococcus* sp. OS5 inoculation. Data are presented as means ± SEM (*n* = 5 biological replicates). Different letters denote significant differences (*P* < .05, ANOVA followed by Tukey’s HSD test).

To further confirm the relationships between OS bacterial community composition and ligninolysis function abundance, we isolated the lignin-degrading bacteria from the OS of *S. litura* and *S. frugiperda* larvae feeding on bok choy, peanut, and maize, using lignin as the sole carbon source (Fig. 5A). A total of 23 strains were obtained. Among them, *B. sedimins* OS20 and *Rhodococcus* sp. OS5 exhibited the highest lignin degradation capacity (ANOVA, *F* = 5.272, *P* < .05; Fig. 5B; Table S2). Consistently, strains OS20 and OS5 were exclusively isolated from the OS of both *S. litura* and *S. frugiperda* when they feeding on bok choy (Table S2). Comparison of the 16S rDNA sequences of OS20 and OS5 against the ASV database reveal that OS20 shared 100% similarity to ASV61, and OS5 100% similarity to ASV123 (Fig. S4). ASV61 and ASV123 were also significantly enriched in the OS of both insect species when bok choy was the host plant (Fig. 5C and D). Subsequently, OS20 and OS5 were inoculated into M9 minimal medium supplemented with alkalie lignin (Fig. 5E). Both strains effectively degraded lignin, achieving degradation rate of 20.98% and 13.29%, respectively, after 10 days (Fig. 5F). Furthermore, the populations of OS20 and OS5 were also increased during the lignin degradation process (Fig. 5G). Finally, OS20 and OS5 were inoculated onto leaf litter fragments within microcosm (Fig. 5H). After 30 days, both strains exhibited dense bacterial aggregation, forming biofilm structures on the litter fragments, which were absent in uninoculated controls (Fig. 5I). Compared to controls, inoculation with OS20 resulted in significant lignin loss in the litter at 30 and 60 days, reducing by 16.95% and 41.01%, respectively. OS5 inoculation reduced the mass of lignin at 30 and 60 days, by 13.85% and 17.62%, respectively (Fig. 5J).

### Genomic and gene expression analyses reveal the lignin-degrading capability of *B. sedimins* OS20 and *Rhodococcus* sp. OS5

**Figure 6:**
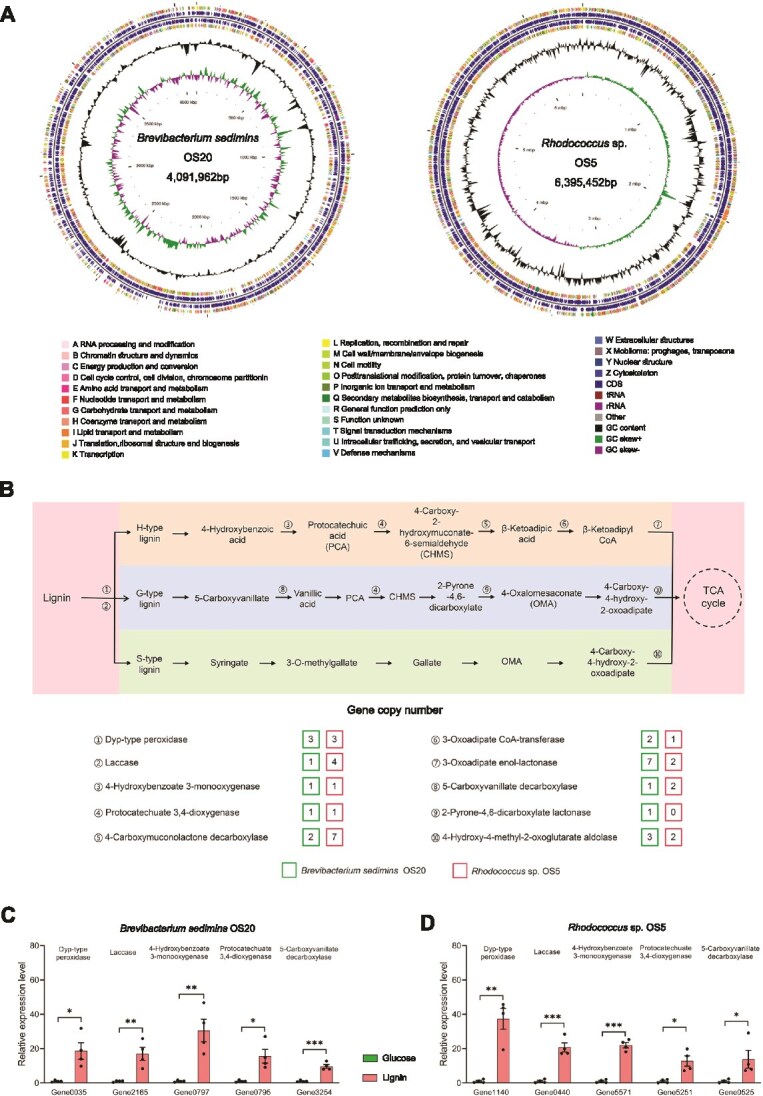
Genomic analysis and lignin degrading-related gene expression of *B. sedimins* OS20 and *Rhodococcus* sp. OS5. (A) Overview of *B. sedimins* OS20 and *Rhodococcus* sp. OS5 genomes. The circles represent (from outside to inside): circles 1–4, genes existing in the genome with different cluster of orthologous groups of proteins (COG) function; circle 5, GC-content; circle 6, GC-skew. Circle 7, genome size. (B) Putative lignin metabolization pathway of *B. sedimins* OS20 and *Rhodococcus* sp. OS5. The pathway was proposed based on genomic data. (C) Expression of genes encoding enzymes involved in lignin degradation in *B. sedimins* OS20 when the strain was cultured with lignin. (D) Expression of genes encoding enzymes involved in lignin degradation in *Rhodococcus* sp. OS5 when the strain was cultured with lignin. Data are presented as means ± SEM (*n* = 4 biological replicates). Asterisks indicate statistically significant differences (^*^P < .05, ^**^P < .01, ^***^P < .001).

To further validate the lignin-degrading capability of *B. sedimins* OS20 and *Rhodococcus* sp. OS5, we sequenced their genomes with a combination of Illumina short read and PacBio long-read technologies. Functional annotation was performed through homology-based search against the NR, GO, KEGG, and CAZy databases to identify genes associated with lignin degradation. The genome of *B. sedimins* OS20 comprised a circular chromosome of 4 091 962 bp with a GC content of 64.62%, containing 3584 open reading frames (ORFs). *Rhodococcus* sp. OS5 contained a circular chromosome of 6 395 452 bp and a plasmid of 652 201 bp, with a total of 6629 ORFs and a GC content of 62.29% (Fig. 6A). Both strains were found to harbor the genes encoding enzymes involving in lignin degradation, particularly of H-type lignin (Fig. 6B). The expression of genes encoding enzymes, such as Dyp-type peroxidase, laccase, 4-hydrobenzoate 3-monooxygenase, protocatechuate 3,4-dioxygenase, and 5-carboxyvanillate decarboxylase was significantly up-regulated in OS20 and OS5 when they were cultured in lignin-containing medium (Fig. 6C and D). Collectively, the genomic and transcriptional data demonstrate that *B. sedimins* OS20 and *Rhodococcus* sp. OS5 are able to degrading lignin.

## Discussion

This study demonstrates that host plant species exert a stronger influence on oral microbial community composition than insect species. Specifically, the oral microbiota of insects feeding on bok choy were significantly distinct from those associated with peanut or maize consumption. Crucially, microbial ligninolysis capacity showed a strong positive correlation with lepidopteran larval performance. This key functional trait was associated with *Brevibacterium* and *Rhodococcus* taxa. Our study indicates that oral microbiomes could be a promising target for biocontrol strategies against lepidopteran pests, including *S. litura* and *S. frugiperda*, which infest high lignin crops like bok choy.

### Plants species shape the diversity and composition of herbivorous insect oral microbial communities

Although our results demonstrated that the host plants exert a stronger influence than insect species on the diversity and composition of the oral microbiota, broader analyses across diverse phytophagous insect taxa are needed to fully clarify the drivers of oral microbiota assembly. More pronounced changes occurred in the oral bacterial community when lepidopteran insects feeding on bok choy, compared to those feeding on peanut and maize. Generally, the insect microbiome is regulated by host physiology and diet [5, 34, 35]. The host physiology encompasses factors, such as pH, oxygen availability, nutritional status, and immunity responses [36, 37]. However, most studies focus on the gut microbiota, despite microbial colonization occurring in other organs including the oral cavity. Compared to the gut, the oral cavity exhibits distinctive features, such as relatively open and oxygen-rich conditions [38]. Notably, it is the site constantly exposed to diverse food particles [39]. Thus, diet is likely the predominant factor governing oral microbiome assembly, either by introducing novel microbes or influencing native oral communities. Diet can act as a source of novel microbes when insects ingested with the food [11, 35, 40]. In the present study, the phyllosphere microbiota from the host plants may contribute to the changes in the oral microbiota. Furthermore, nutritional properties of the food can also shape the insect’s oral microbiome via their effect on the resident microbial populations.

### Ligninolysis within the oral microbial community is associated with lepidopteran herbivore performance

Food quality not only alters the diversity and composition of oral microbiota, but also shapes its functional profile [15]. Specially, microbial ligninolysis capacity exhibited significant and positive correlations with both leaf lignin content and larval performance. This indicates that leaf lignin drives functional adaptation within the oral microbial community. Notably, ligninolysis was enriched in the oral microbiota of *S. litura* and *S. frugiperda* when feeding on bok choy, which corresponds to the higher lignin content in bok choy leaves. While lignin is classically regarded as a plant defensive compound that fortifies cell walls against chewing herbivores [41, 42]. Moreover, most herbivorous insects, including *S. litura* and *S. frugiperda*, lack inherent lignin-degrading enzymes [43]. Consequently, these insects utilize their microbiota to metabolize lignin into key energy sources to support growth. This mediation of a plant defense compound highlights the role of microbes in plant-insect coevolutionary dynamics.

Most of the predicted functional traits within the oral microbial communities were associated with nitrogen metabolism, a pattern also observed in gut microbial communities [44]. Nitrogen is widely considered as the most limiting nutrient for herbivorous insect growth and development [9, 45]. To overcome this limitation, insects form a symbiotic relationship with microbes capable of nitrogen metabolism, acquiring nitrogen through biological nitrogen fixation and nitrogenous waste recycling [46–48]. Consistently, we observed enrichment of nitrogen fixation, nitrate reduction, nitrate respiration, nitrite respiration, nitrate denitrification, nitrate denitrification, and ureolysis pathways in the oral microbiota. Additionally, plant pathogen-associated functions were also enriched, suggesting the potential direct interactions between oral microbes and host plants. Previous studies reported that chewing insects deposit oral bacteria into wounds created on leaves to suppress plant immune responses and enhance feeding efficiency [5, 6, 16].

### *Brevibacterium sedimins* OS20 and *Rhodococcus* sp. OS5 degrade lignin

Using lignin as the sole carbon source, we isolated two highly efficient lignin-degrading strains, *B. sedimins* OS20 and *Rhodococcus* sp. OS5, from the OS of insects feeding on bok choy. Although there have been no reports concerning the degradation of lignin by *B. sedimins*, this genus demonstrates metabolic capabilities for phenolics, polycyclic aromatic hydrocarbons, and polystyrene [49–51]. The degradation pathways involve various enzymes, such as cytochrome P450 monooxygenases/dioxygenases and laccases, which were also implicated in lignin breakdown [52–54]. This supports the lignin-degrading potential of *B. sedimins* OS20. Consistently, the genome of *B. sedimins* OS20 was found to contain genes encoding enzymes involved in lignin degradation, such as Dyp-type peroxidase and laccase. Dyp-type peroxidases are heme-containing enzymes present in fungi and bacteria, known for their ability to degrade recalcitrant anthraquinone dyes and lignin [55]. Interestingly, *B. sedimins* is commonly found as a plant rhizobacterium, suggesting that insects may acquire this bacterium from the environment during feeding on host plants [56]. *Spodoptera litura* and *S. frugiperda* larvae exhibit strong circadian behavior, feeding at night, and sheltering in soil during daytime [57]. Studies have indicated that herbivorous insects actively recruit soil- or plant-derived microbes as oral or gut symbionts, to enhance feeding efficiency [58]. *Rhodococcus* species metabolize lignin or its derivatives as carbon sources into lipids [59, 60]. Lipids are essential for insect growth and development [61]. Consistent to other studies, Dyp-type peroxidase was also found in the *Rhodococcus* sp. OS5. Moreover, *Rhodococcus* species commonly act as commensals in insect guts [62]. These findings suggest lignin degradation may occur through the synergistic interplay of the oral-gut microbiota axis.

## Conclusion

In this study, we present evidence that plant species influence the diversity and composition of the oral microbial community. The oral microbial ligninolysis capacity is positively correlated with herbivorous insect performance. Although the lignin degradation mechanisms employed by *Brevibacterium* and *Rhodococcus* spp. require further elucidation, our findings reveal the critical contribution of specific oral microbial taxa to insect fitness through the breakdown of recalcitrant plant compounds. Identifying such functional taxa is essential for developing novel biocontrol strategies and advancing sustainable agriculture. Furthermore, our work highlights insects as reservoirs of microbial resources with significant biotechnological potential. Ultimately, our research advances our understanding of plant–insect–microbe interactions, emphasizing the complex multitrophic relationships underlying coevolutionary dynamics.

## Supplementary Material

Supplementary_Materials_ycaf229

## Data Availability

The raw data of oral microbial amplicon reads have been deposited in the National Center for Biotechnology Information (NCBI) Sequences Reads Archive (SRA) database under accession code BioProject PRJNA1285817. The genome of *Brevibacterium sedimins* OS20 and *Rhodococcus* sp. OS5 used in this study is available in the NCBI Genome database under accession code BioProject PRJNA1355309 (OS20) and PRJNA1355319 (OS5). The data supporting the findings of this study are provided in the manuscript and its supplemental data.
