# Supplementary material for: Oral microbiome is related to lepidopteran herbivore performance by lignin degradation
Source: ISME Commun. 2025 Dec 6;5(1):ycaf229. doi: 10.1093/ismeco/ycaf229 (PMC12753300; doi:10.1093/ismeco/ycaf229)
Supplement: Supplementary_Materials_ycaf229 [file supplementary_materials_ycaf229.docx]

Supplementary Materials for

**Oral microbiome is related to lepidopteran herbivore performance by lignin degradation**

**Supplementary Figures**

**
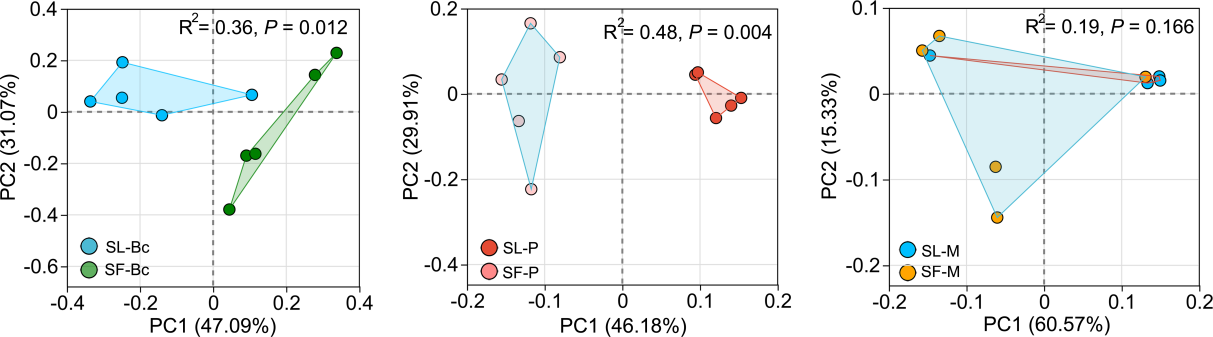
**

**Supplementary Figure 1.** Principal Coordinates Analysis (PCoA) based on Bray-Curtis distance calculated from the relative abundance of bacterial ASVs in OS samples. Statistical significance was assessed by permutational multivariate analysis of variance (PERMANOVA).

**
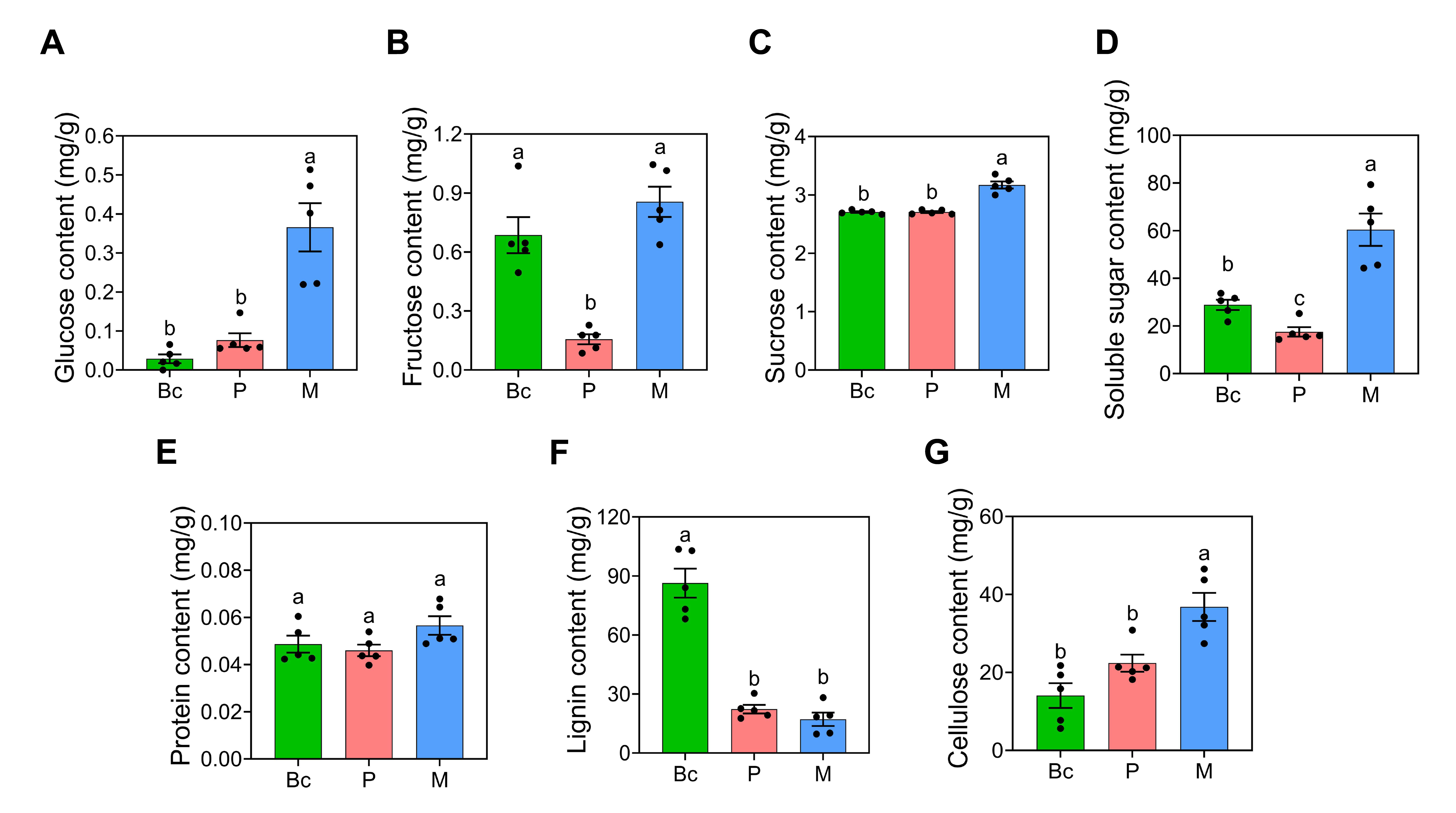
**

**Supplementary Figure 2.** Leaf chemistry properties. The content of (A) glucose, (B) fructose, (C) sucrose, (D) soluble sugar, (E) protein, (F) lignin and (G) cellulose in bok choy, peanut, and maize leaves. Data are presented as means ± SEM (n = 5 biological replicates). Different letters denote significant differences (*P* < 0.05, ANOVA followed by Tukey’s HSD test).


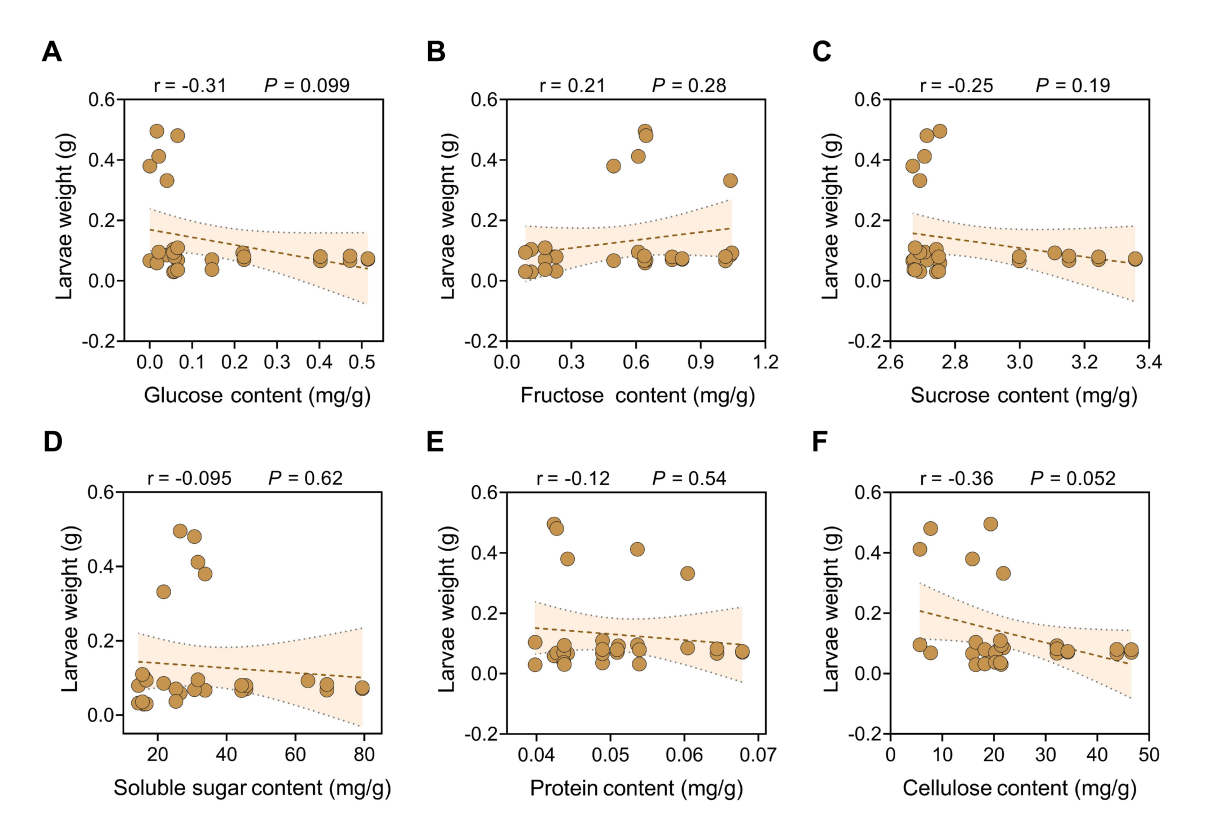


**Supplementary Figure 3.** Leaf soluble sugars, protein, and cellulose content in relation to larval weight. (A) Leaf glucose content in relation to larval weight. (B) Leaf fructose content in relation to larval weight. (C) Leaf sucrose content in relation to larval weight. (D) Leaf soluble sugar content in relation to larval weight. (E) Leaf protein in relation to larval weight. (F) Leaf cellulose content in relation to larval weight.


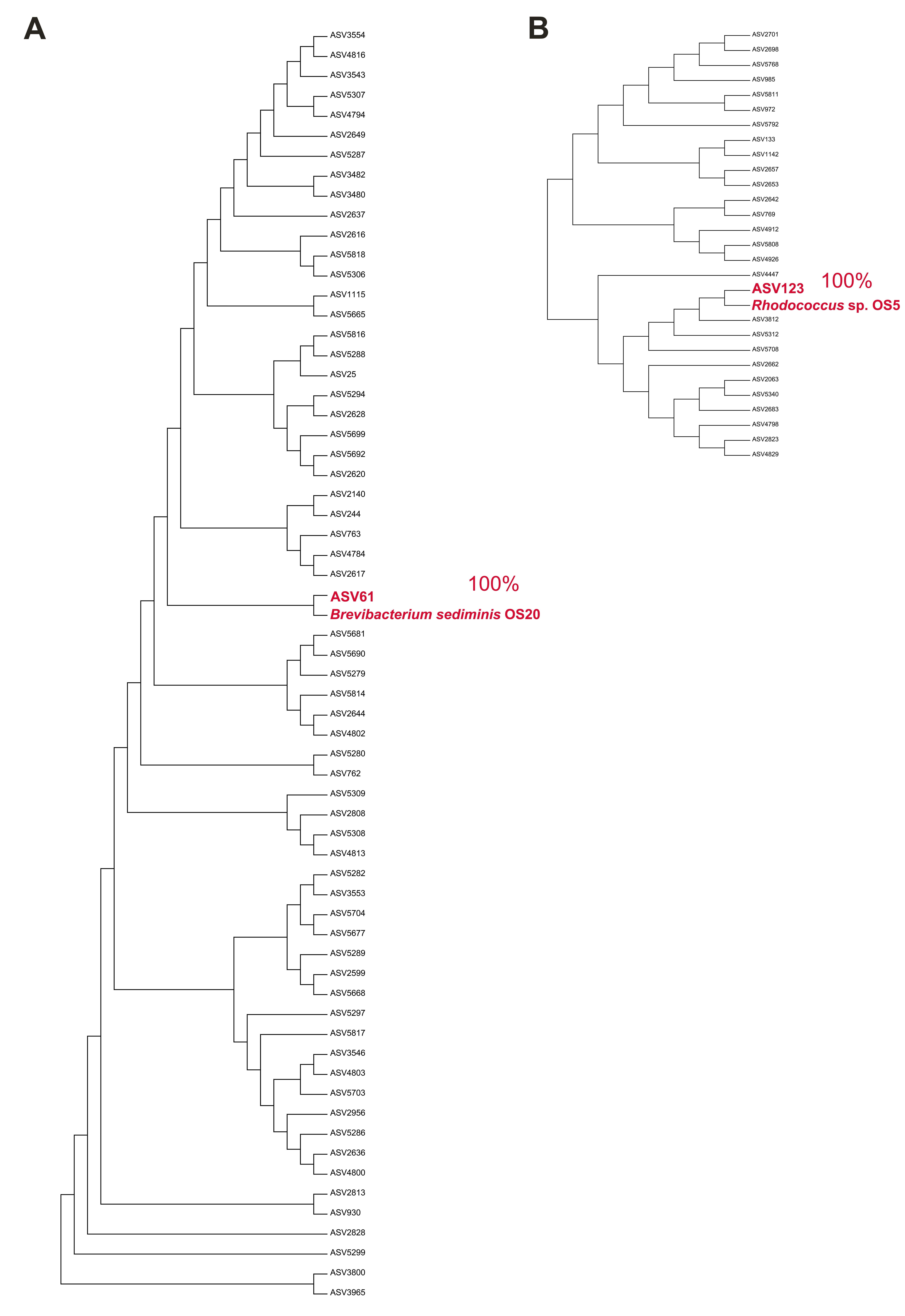


**Supplementary Figure 4.** (A) Phylogenetic reconstructions of *Brevibacterium sedimins* OS20 and ASV61. (B) Phylogenetic reconstructions of *Rhodococcus* sp. OS5 and ASV123.

**Supplementary Tables**

**Supplementary Table S1.** Primers used for qRT-PCR analysis

| **Gene ID** | **Primer Forward** | **Amplicon size (bp)** | **Annotation** |
| --- | --- | --- | --- |
| *Brevibacterium sedimins* OS20 | | | |
| Gene0035 | GACCGTCTGGCAACAGGCATTC | 129 | Dyp-type peroxidase |
|  | AGGAAGTCCTTGGCTCTGCTCTC |  |  |
| Gene2165 | GGACTCGGATTGGCGATCATGG | 130 | Laccase |
|  | CCACTGTTCTGCCGACGATTCC |  |  |
| Gene0797 | ACTTCCGACGGCGACGAACT | 189 | 4-Hydroxybenzoate 3-monooxygenase |
|  | GGAGCGGGAGTAGATGAGGACTTC |  |  |
| Gene0795 | GTCCCCGATGCCATTCTTGA | 253 | Protocatechuate 3,4-dioxygenase alpha subunit |
|  | CCTCAGGCAGATAGATGCGG |  |  |
| Gene3254 | CCCTGACTTACCCGCACAAA | 338 | 5-Carboxyvanillate decarboxylase |
|  | CCGTGGGAACTTGGACAGAA |  |  |
| Reference gene | ACGGCCCAGACTCCTACG | 200 | 16S rRNA [1] |
|  | CGCACCCTACGTATTACCG |  |  |
| *Rhodococcus* sp. OS5 | | | |
| Gene1140 | ATGCTGTGCGAGTTCTGACCAAG | 190 | Dyp-type peroxidase |
|  | CCGTCGTGCCAGATCAGTGAATC |  |  |
| Gene0440 | GGTGGTGTTCCGTCGCAACT | 150 | Laccase |
|  | CGTCAGGTTGTTCAGCGTCATCT |  |  |
| Gene5571 | GCACTGGCGAAGGCAATGGA | 144 | 4-Hydroxybenzoate 3-monooxygenase |
|  | GTCTGGCAAGCGATGGAGCAT |  |  |
| Gene5251 | ACAAGACGACTTCCCTTCGC | 380 | Protocatechuate 3,4-dioxygenase alpha subunit |
|  | GTGGTTCATCCACGGGTAGG |  |  |
| Gene0525 | GCTGAAGGTCGAGTCGGAAA | 560 | 5-Carboxyvanillate decarboxylase |
|  | CGATCTTGTCGAACACCCCA |  |  |
| Reference gene | TCGTGAGATGTTGGGTTAAG | 210 | 16S rRNA [2] |
|  | CCTCTGTACCGGCCATTGTAG |  |  |

**Supplementary Table 2.** Identification of OS bacteria isolates.

| **Strain** | **Accession number** | **Source** |
| --- | --- | --- |
| Staphylococcus saprophyticus OS1 | PV991484 | SF-Bc |
| Paenibacillus sp. OS2 | PV991485 | SF-Bc |
| Micrococcus antarcticus OS3 | PV991486 | SF-Bc |
| Janibacter sp. OS4 | PV991487 | SF-Bc |
| Rhodococcus sp. OS5 | PV991488 | SF-Bc |
| Staphylococcus xylosus OS6 | PV991489 | SF-Bc |
| Paenibacillus mobilis OS7 | PV991490 | SF-M |
| Priestia megaterium OS8 | PV991491 | SF-M |
| Staphylococcus capitis OS9 | PV991492 | SF-M |
| Agrococcus citreus OS10 | PV991493 | SF-M |
| Rhodococcus sp. OS11 | PV991494 | SF-P |
| Dermacoccus sp. OS12 | PV991495 | SF-P |
| Microbacterium hydrothermale OS13 | PV991496 | SF-P |
| Serratia marcescens OS14 | PV991497 | SL-Bc |
| Enterococcus mundtii OS15 | PV991498 | SL-P |
| Staphylococcus sp. OS16 | PV991499 | SL-P |
| Brucella pseudogrignonensis OS17 | PV991500 | SL-P |
| Paenibacillus taichungensis OS18 | PV991501 | SL-M |
| Micrococcus luteus OS19 | PV991502 | SL-M |
| Brevibacterium sediminis OS20 | PV991503 | SL-Bc |
| Enterococcus sp. OS21 | PV991504 | SL-M |
| Enterococcus casseliflavus OS22 | PV991505 | SL-M |
| Enterococcus gallinarum OS23 | PV991506 | SL-M |

**Supplementary references**:

1. Majtan T, Halgasova N, Bukovska G, et al. Transcriptional profiling of bacteriophage BFK20: coexpression interrogated by "guilt-by-association" algorithm. *Virology* 2007;359:55–65. <https://doi.org/10.1016/j.virol.2006.09.028>
2. Zampolli J, Orro A, Manconi A, et al. Transcriptomic analysis of Rhodococcus opacus R7 grown on polyethylene by RNA-seq. *Sci Rep* 2021;11:21311. https://doi.org/10.1038/s41598-021-00525-x
